# Longitudinal Trends and Risk Factors for Depressed Mood Among Canadian Adults During the First Wave of COVID-19

**DOI:** 10.3389/fpsyt.2021.666261

**Published:** 2021-07-16

**Authors:** Gustavo S. Betini, John P. Hirdes, Rhéda Adekpedjou, Christopher M. Perlman, Nathan Huculak, Paul Hébert

**Affiliations:** ^1^School of Public Health and Health Systems, University of Waterloo, Waterloo, ON, Canada; ^2^Centre de Recherche CHUM (Centre Hospitalier de l'Université de Montréal), Montréal, QC, Canada; ^3^Canadian Red Cross, Montreal, QC, Canada; ^4^Department of Medicine, Université de Montréal, Centre de Recherche CHUM (Centre Hospitalier de l'Université de Montréal), Montréal, QC, Canada

**Keywords:** mental health care, depression mood, anxiety, general population, coronavirus–COVID-19

## Abstract

**Background:** The COVID-19 pandemic has raised serious concerns about the mental health impact of people directed and indirectly affected by the virus. Because this is a rapidly evolving situation, our goal was to explore potential risk factors and trends in feelings of anxiety and depression among the general population in Canada over the first 5 months of the pandemic.

**Methods:** We completed on-line surveys of 3,127 unique individuals representative of the Canadian general population at 4 discreet periods every 6 weeks from April 15th to July 28th 2020. We assessed feelings of anxiety, depression and loss of interest with the interRAI self-reported mood scale using a multivariable generalized estimating equation model to examine factors associated with having a 5+ score on the scale (indicating potentially depressed mood). We also investigated potential longitudinal trends to examine temporal variation in mood scores.

**Results:** More than 30% of participants felt highly anxious, depressed, and disinterested in everyday activities in the first survey (April), but this number decreased to about 20% over 4 months. Feeling lonely, younger age, feeling overwhelmed by one's health needs, having financial concerns, and living outside of Québec were significantly associated with depressed mood.

**Interpretation:** The prevalence of depressed mood during the pandemic was between 2 and 3 times the pre-pandemic rate (especially among young people), but it can change rapidly in response to social changes. Thus, monitoring of psychological distress among vulnerable groups that may benefit from additional supports should be a priority.

## Introduction

There has been a growing concern that, without focused mitigation efforts, the COVID-19 pandemic has the potential to increase mental health problems worldwide (1–12). In addition to fear of contracting COVID-19, lock-downs, uncertainty, self-isolation and social distancing are disrupting everyday lives, creating personal, social and economic challenges with potential negative psychological effects (10, 11) despite the fact that public health guidelines, such as face mask use, can have positive effects on stress and anxiety (3). Quarantine has been reported to cause post-traumatic stress symptoms, confusion, and anger with potentially long lasting effects (4) and depression and anxiety were estimated to be higher among quarantined individuals during the initial stages of COVID-19 in China (10). In addition, risk factors for mental health problems during COVID-19 are reported to include female gender, younger age, presence of chronic and psychiatric illnesses, unemployment, student status, and frequent exposure to social media or news concerning the pandemic (5–10). This means that the general population can also be adversely affected by mental health consequences of pandemics and mental health considerations should be taken into account in addition to the physical effects of the virus (4, 10, 13).

Although these studies provide good evidence of the importance of understanding the mental health impact of COVID-19 on the general population, it is still not clear how these negative effects might change with the dynamics of COVID-19 and the changes in the public health policies aiming to contain its spread. This is important because most of these studies were conducted as cross-sectional snapshots at varying time periods of the pandemic, making comparison among studies difficult (10). The objective of this study is to examine the mental health impacts of COVID-19 as well as longitudinal changes in the general Canadian population.

## Methods

### Web-Based Survey

We conducted longitudinal web-based interviews with the general adult population in Canada from April to July in four discrete surveys, 4–6 weeks apart from each other (Table 1). We used a professional polling company to obtain a sample that was representative of the Canadian population (Table 1) when applying survey weights. Participants were recruited via phone (60%), invitation (25%), social media (5%), offline recruitment (5%), partnerships and campaigns (5%). Among the 3,127 participants, ~80% were present in two or more surveys and 1,510 (66%) were present in all surveys (Table 1). Mental health status was assessed with three questions from the interRAI self-reported mood scale, which assesses levels of anxiety, depression, and loss of interest (14). The questions were: “In the last 3 days, how often have you felt: (a) anxious, restless, or uneasy, (b) sad, depressed, or hopeless and (c) little interest or pleasure in things you normally enjoy.” Each item has scores ranging from 0 (not present) to 3 (daily), and scores for the three items are summed to create a scale with a value between 0 and 9. Higher scores representing more frequent and varied mood symptoms (Cronbach's alpha = 0.81). We set a threshold for having substantially depressed mood at 5 or more based on previous analyses that indicate this threshold to be associated with suicide-related ideation in community mental health populations (results available on request). Socio-demographic variables and main concerns before and during the pandemic (e.g., financial concerns, food insecurity levels, and loneliness) were also assessed during the interviews (Tables 1, 2).

**Table 1:** Profile of the participants in each survey.

	**Survey 1 April 15–20th**	**Survey 2 May 6–13th**	**Survey 3 June 3rd−9th**	**Survey 4 July 22–28th**
N^*^	2,200	2,264 (314)	2,280 (352)	2,201 (241)
Recontacts in previous survey	na	86%	84%	87%
**Gender**				
Male	48%	48%	49%	49%
Female	52%	52%	51%	51%
**Age**				
18–34	27%	27%	26%	24%
35–54	34%	34%	36%	37%
55–64	17%	17%	17%	17%
65–74	12%	12%	12%	12%
75+	9%	9%	9%	9%
**Province**				
British Columbia	14%	14%	14%	14%
Alberta	11%	11%	11%	11%
Manitoba/Saskatchewan	6%	6%	7%	7%
Ontario	38%	38%	38%	38%
Quebec	23%	23%	23%	23%
Atlantic	7%	7%	7%	7%
**Region**				
Quebec	23%	23%	23%	23%
Rest of Canada	77%	77%	77%	77%
**Area type**				
Urban	88%	89%	90%	90%
Rural	12%	11%	10%	10%
**Mother tongue**				
French	21%	20%	20%	20%
English	67%	66%	66%	65%
Other languages	12%	13%	14%	14%
**Ethnic origin**				
Caucasian (White)	83%	81%	81%	80%
Aboriginal/First nations	1%	1%	1%	1%
Black	2%	2%	2%	2%
Chinese	3%	5%	5%	5%
Other	9%	10%	10%	10%
**Children in the household**				
Yes	28%	28%	28%	28%
No	72%	72%	72%	72%
**Living situation**				
Alone	20%	20%	21%	21%
With spouse (partner only)	32%	31%	32%	32%
With spouse/partner and other(s)	27%	27%	26%	26%
With child(ren) (no spouse/partner)	5%	5%	6%	6%
With parent(s) or guardian(s)	9%	11%	10%	10%
With sibling(s)	1%	1%	1%	1%
With other relative(s)	2%	2%	2%	2%
With nonrelative(s)	3%	3%	3%	3%
**Vulnerable senior**				
Yes	na	2%	2%	2%
No	na	98%	98%	98%
**Education**				
Elementary/High school	33%	31%	32%	30%
College	40%	41%	41%	43%
University	27%	27%	27%	27%
**Occupation**				
Office/services/sales	na	23%	22%	23%
Manual worker	11%	10%	10%	9%
Professional	19%	19%	20%	20%
Homemaker	3%	4%	4%	4%
Student	7%	7%	7%	6%
Retired	27%	27%	28%	28%
Unemployed	5%	5%	5%	4%

* *Number of participants added to each survey: 314, 352, and 241 in surveys 2, 3, and 4, respectively*.

**Table 2:** The association between score of 5+ on self-reported mood scale and a number of risk factors.

	**Bivariate analysis unadjusted OR**			**Multivariable analysis adjusted OR**		
	**OR**	**95% CI**	***P*-value**	**OR**	**95% CI**	***P*-value**
**Age (years)**			**< 0.001**			**< 0.001**
18–24 vs. ≥ 65	5.61	3.43–9.17	<0.001	2.62	1.58–4.32	<0.001
25–34 vs. ≥ 65	3.52	2.37–5.22	<0.001	1.96	1.21–3.17	0.006
35–44 vs. ≥ 65	3.39	2.28–5.03	<0.001	2.67	1.73–4.14	<0.001
45–64 vs. ≥ 65	2.31	1.69–3.15	<0.001	1.88	1.30–2.72	<0.001
**Gender**			**0.004**			
Female vs. Male	1.49	1.13–1.95	0.004			
**Province**			**< 0.001**			**0.01**
Others vs. Quebec	2.1	1.49–2.94		1.64	1.11–2.43	
**Language**			**< 0.001**			
English vs. French	1.89	1.33–2.69	<0.001			
Bilingual vs. French	3.21	1.59–6.47	0.001			
Others vs. French	1.90	1.08–3.35	0.03			
**Education**			0.70			
College vs. Elementary/High school	0.94	0.66–1.33	0.73			
University vs. Elementary/High school	1.07	0.77–1.47	0.69			
**Living with**			**< 0.001**			
Spouse/partner vs. Alone	0.75	0.52–1.08	0.12			
Parent/guardian vs. Alone	1.99	1.16–3.40	0.01			
Other relatives vs. Alone	1.23	0.86–1.76	0.25			
Non-relatives vs. Alone	2.16	0.98–4.77	0.06			
**Occupation**			**< 0.001**			
Homemade vs. Manual worker	0.94	0.41–2.12	0.88			
No answer vs. Manual worker	5.33	1.46–19.49	0.01			
Retired vs. Manual worker	0.43	0.32–0.57	<0.001			
Student vs. Manual worker	1.78	1.04–3.04	0.04			
Unemployed vs. Manual worker	1.31	0.69–2.49	0.42			
**Ethnicity**			0.07			
Caucasian vs. Other	1.01	0.69–1.48	0.94			
No answer vs. Other	3.49	1.17–10.43	0.03			
**Health before Covid19**			**< 0.001**			
Fair vs. Excellent/good	1.93	1.35–2.80	0.003			
Poor vs. Excellent/good	3.81	1.57–9.26	<0.001			
**Health in the past month**			**< 0.001**			
Fair vs. Excellent/good	2.73	1.98–7.36	<0.001			
Poor vs. Excellent/good	6.15	3.01–12.52	<0.001			
**Family overwhelmed by one's health needs**						<0.001
Yes vs. No	4.87	3.13–7.56	<0.001	3.92	2.39–6.41	
**Feel lonely**			**< 0.001**			**< 0.001**
Only in specific situations/I do not feel lonely	2.84	1.75–4.62	**< 0.001**	2.20	1.31–3.70	0.003
Occasionally/I do not feel lonely	4.74	3.1–7.22	**< 0.001**	3.80	2.45–5.91	<0.001
Daily/I do not feel lonely	21.31	13.66–33.26	**< 0.001**	16.65	10.49–26.42	<0.001
**Financial status–difficulty making ends meet**			**< 0.001**			
Before and after COVID-19 vs. None	3.37	2.45–4.64	<0.001	1.93	1.35–2.77	0.003
Before or after COVID-19 vs. None	2.39	1.70–3.37	<0.001	1.69	1.11–2.58	0.02
**Have a way–get food and medication**			**< 0.001**			
Yes vs. No	2.77	1.65–4.66	**< 0.001**			
**Feel safe and comfortable in home**			**< 0.001**			
Sometimes vs. Never	0.56	0.20–1.56	0.27			
Always vs. Never	0.12	0.05–0.32	<0.001			
**Have people–count on**			**< 0.001**			
Sometimes vs. Never	0.54	0.31–0.96	0.04			
Always vs. Never	0.27	0.16–0.44	<0.001			
**Can get help if needed**			**< 0.001**			
Sometimes vs. Never	0.53	0.32–0.87	0.01			
Always vs. Never	0.23	0.15–0.36	<0.001			

*Odds ratio were calculated with a bivariate (unadjusted) and multivariable (adjusted) analysis in survey 1 (April 10–15th)*

*Bold values represent significant values at α = 0.05*.

### Statistical Analysis

**Figure 1:**
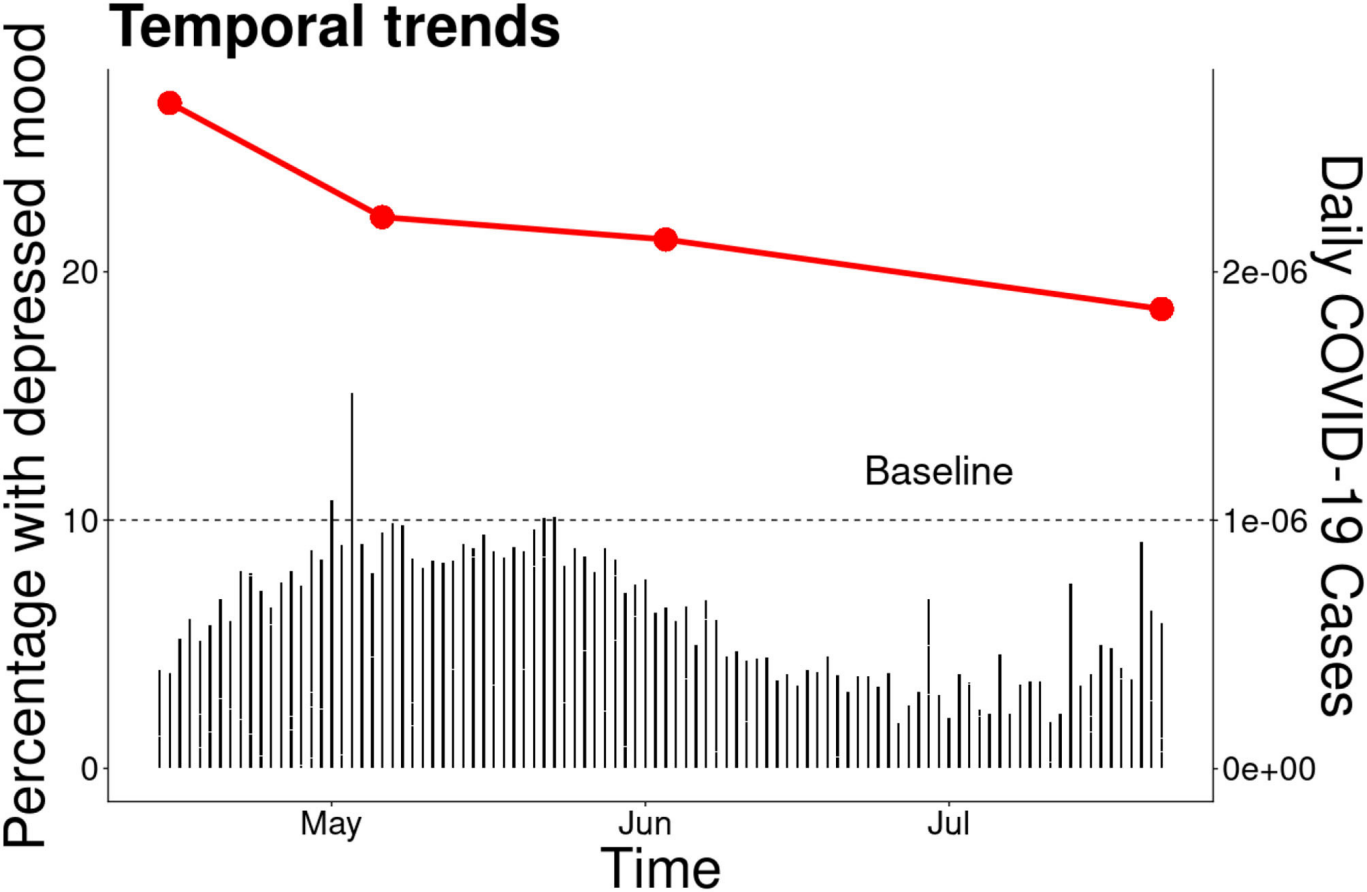
Longitudinal trends in percentage of participants with 5+ score on the self-reported mood scale by study survey.

To understand the risk factors associated with the mental health impact of COVID-19, we used a bivariate regression with the percentage of respondents with a 5+ score in the self-reported mood scale as response variable and socio-demographic factors as explanatory variables (Tables 1, 2). This threshold has been shown to be associated with clinical depression and self-harm ideation in community mental health populations (results available on request). We compared Quebéc with the rest of Canada because of known differences in mental health state of populations of these two geographic regions (15, 16), which may be a result of cultural differences (i.e., Québec is mainly francophone while the rest of Canada is predominantly anglophone). For simplicity, we only used data from survey 1 for an initial logistic regression model given that the levels of depression, anxiety and loss of interest were stronger at this stage (Figure 1). We then examined a longitudinal interaction between age and survey in a generalized estimating equation model to investigate potential temporal trends in the mental health impact among different age groups. We focused on age because of the great physical health burden that COVID-19 has on older adults (10, 17). We did not find a significant interaction between age and survey wave. Therefore, we presented the model with main effects only. We weighted all analysis using the survey weights to match the sample to population distributions in the latest Statistics Canada census according to gender, age, region, education, mother tongue, living arrangements, and presence of children in the household. We used data from a general population survey done in the Waterloo Region in 2011 (data available upon request) to compare our results with a base level of the same indicators for the general population before the beginning of the COVID-19 pandemic. The baseline level of scores of 5+ on the self-reported mood scale in those surveys ranged between 6.5% in 2011, which is comparable to anxiety (6.3–50.9%, including mild to severe levels) and depression levels (3.6–7.2%) reported in other studies conducted before the pandemic (18). To provide contextual information, daily COVID-19 cases (Figure 1) were obtained from Berry et al. (19) and figures were produced with the *ggplot2* package in R (20).

## Results

We found that up to 44.3% of the participants had substantial level of depressed mood based on indicators of anxiety, depression and loss of interest in the April survey. Only education and ethnicity were not significant risk factors in the bivariate analysis (Table 2). The final multivariable model indicated that age [*F*_(4, 2216)_ = 7.26, *P* < 0.001], province [*F*_(1, 2219)_ = 6.14, *P* = 0.013], feeling overwhelmed by one's health needs [*F*_(1, 2219)_ = 29.56, *P* = 0.001], loneliness [*F*_(1, 2217)_ = 52.37, *P* < 0.0001], and financial concerns [*F*_(1, 2218_ = 7.13, *P* < 0.001] were significantly associated with depressed mood [*F*_(11, 2209)_ = 56.74, *P* < 0.001; c-statistics = 0.836]. The odds of having a depressed mood were 2.62 times higher in young (18–24) than older adults [65+; 95% confidence interval (CI) 1.58–4.32, *P* < 0.001], 1.64 times higher in people from other provinces compared to Québec (95% CI 1.11–2.43, *P* = 0.013), 3.92 times higher in people who felt overwhelmed by their health needs (95% CI 2.39–6.41, *P* < 0.001), 16.65 times higher in people who felt lonely daily compared to those that did not feel lonely (95% CI 10.49–26.42, *P* < 0.0001) and 1.93 times higher in people that had financial concerns before and after the pandemic than people without financial concerns (95% CI 1.35–2.77, *P* < 0.001; Figure 1 and Table 2). We found a significant temporal trend in the generalized estimating equation model, suggesting a decrease in the odds of depressed mood over time compared with the initial stage of the survey done in April 2020 (Figure 1 and Table 3).

**Table 3:** The association between the self-report mood scale score of 5+ and a number of risk factors over four study waves.

**Parameter**	**Estimate**	**95% CI**		***Z***	***P*-value**	**Adjusted OR**	**95% CI**	
**Intercept**	−2.761	−3.126	−2.398	−14.87	<0.001			
**Age group (ref = 18–24)**								
25–34	0.12	−0.20	0.45	0.74	0.45	1.13	0.82	1.56
35–44	−0.23	−0.58	0.12	−1.28	0.20	0.79	0.56	1.13
45–64	−0.25	−0.54	0.03	−1.73	0.08	0.78	0.58	1.03
65+	−0.79	−1.07	−0.51	−5.56	<0.001	0.45	0.34	0.60
**Province (ref = Quebec)**								
Others	0.26	0.05	0.46	2.46	0.01	1.30	1.05	1.59
**Health status (ref = no concerns)**								
Overwhelmed by one's health needs	1.33	1.05	1.62	9.16	<0.001	3.80	2.85	5.05
**Loneliness (ref = not lonely)**								
Lonely only in specific situations	0.74	0.45	1.03	4.94	<0.001	2.09	1.56	2.80
Lonely occasionally	1.39	1.14	1.64	10.83	<0.001	4.02	3.13	5.17
Lonely daily	2.96	2.69	3.22	22.11	<0.001	19.29	14.84	25.07
**Financial status (ref = no worries)**								
Financial worry before and after COVID-19	0.69	0.48	0.89	6.56	<0.001	2.00	1.62	2.45
Financial worry before or after COVID-19	0.62	0.38	0.86	5.09	<0.001	1.86	1.46	2.35
**Study wave (ref = Survey 1)**								
Survey 2	−0.23	−0.46	0.01	−1.89	0.06	0.80	0.63	1.01
Survey 3	−0.27	−0.52	−0.03	−2.20	0.03	0.76	0.60	0.97
Survey 4	−0.32	−0.57	−0.07	−2.52	0.01	0.72	0.56	0.93

*Odds ratio were calculated with a longitudinal multivariable generalized estimating equation model*.

## Discussion

### Interpretation

Our study provided a longitudinal view of the mental health impact of COVID-19 on about 3,000 participants followed over a 4-month period. The impact was most pronounced on the mental health of younger Canadians and those who reported feeling lonely. In addition, the odds of serious mood disturbance were strongest at the beginning of the pandemic (April), with a rapid decrease from April to July. However, the absolute levels in July were still 2 times higher compared to the pre-pandemic levels. Although there appears to be potential for resilience and fast recovery in part of the population, the absence of complete recovery could result in even higher levels of anxiety and depression during new waves of the pandemic.

Our results are in line with other studies on the mental health effects of COVID-19, both in the strength of the association and key risk factors (7, 10, 21–23). A meta-analysis found that during the pandemic, prevalence of depression symptoms was 33.7% (95% confidence interval 27.5–40.6) and 31.9% for anxiety [95% CI 27.5–36.7; (10)]. As in our study, these levels were, in average, higher than the pre-pandemic levels we noted in earlier studies based on our measure (between 6.5 and 9.7%) as well as comparable rates reported by others (10). Other longitudinal studies have reported a mix of results, with small increases in feelings of depression and decreased anxiety (24) or no trend overall (25). We showed roughly a 30% decrease in the odds of disturbed mood spanned a 4-month period, whereas other longitudinal studies investigated trends over a much shorter time period (24, 25). Some of the discrepancies reported in the published literature could be explained by the phase of the pandemic when the study was conducted.

We also observed that young respondents were among the most affected groups, even after controlling for potential confounding factors such as employment status, gender, health, and economic status. This may reflect a true age-group difference in the COVID-19 experience, but it may also reflect generational differences in comfort related to reporting mental health symptoms. Student status and gender have been identified as risk factors in other studies (10, 26), but these were only significant in our bivariate model. Although student status was strongly associated with age, which may explain it not persisting as a predictor of mood disturbance in the multivariable model, it is less clear why gender was not a significant risk factor when we controlled for other sociodemographic variables.

Although the levels of anxiety and depression reported here are in line with the published literature (10, 17), COVID-19 happened during a time of changing public sentiment about global political, economic, and climate stability with increased focus on natural disasters like wild fires (e.g., bushfires in Australia and California), flooding (e.g., Fort McMurray, Canada), and an increase in public protest related to racial equality. These events might be local and sometimes outside of Canada, but the coverage was widespread and persistent in the media at the time they occurred, which may have negatively affected mental health of Canadians (27). Thus, it is unclear from our results whether the pandemic was the primary driver of changes in mood that we observed or it was only one of the many contributors, potentially acting as an amplifier of these other source of stress (28).

The negative effects of COVID-19 on the mental health we observed are in agreement with studies conducted in other countries. However, the pandemic also brought some opportunities to improve mental health services via virtual care. For example, a recent meta-analysis strongly suggest that digital cognitive behavioral therapy for insomnia is highly effective (29) and telemedicine and virtual software can help to stop the spread of COVID-19, decrease the use of hospital resources while treating patients (30). Therefore, virtual care could improve the accessibility of treatments even during lockdowns and potentially increase the use of these services after the pandemic.

We observed clear trends on mental health indicators on a period of 4 months, but we still do not know what the long-term consequences of COVID-19 will be nor what policies will successfully mitigate its mental health impacts. Information on the long-term impact of past pandemics, such as the Spanish Flu, is scarce. However, some studies reported that people developed psychiatric disorders several years after the 2003 SARS-CoV-1 pandemic (31). Moreover, studies on natural disasters, such as hurricane, fires and earthquake also point to long-term effects where lifetime post-traumatic stress disorder rates can be up to 40% higher in disaster survivors compared to controls (32, 33). Finally, studies have shown that the pandemic have exacerbated racial, social, and economic disparities (34, 35), which could also persist for many years after the pandemic. Given the substantially elevated levels of distressed mood compared with pre-COVID-19 levels, it is important to monitor whether long-term mental health effects persist in the general population.

### Limitations

Our overall response rate was in the 35% range and the assembled sample was representative of Canadians. In addition, by repeatedly sampling more than 80% of the same individuals over time, we are confident that temporal trends were accurately measured. Despite this, vulnerable groups may not have been well represented. For instance, older socially isolated adults, persons in facility-based settings (e.g., long-term care), and marginalized groups may not have internet access or may not be able to participate because of other barriers (36–38). In addition, depressive symptoms tend to decrease with age (39, 40). Thus, the absolute values of our mental health indicators might be biased, specifically among older adults (65+), despite the fact that socio-demographic factors were weighted in the statistical analysis. Fortunately, non-response to surveys does not substantially harm the ability to estimate associations among variables including to investigate temporal trends (36). Another limitation in our web-survey approach is the small subsample sizes of minority groups who may be deferentially affected by mental health concerns. Future efforts to examine the impact of COVID-19 on race and ethnicity should over-sample minority groups to allow for adequate subsample sizes.

## Conclusions

In our survey, we showed that the pandemic increased feelings of anxiety, depression, and loss of interest symptoms 2–3-fold, especially in young people. We also documented that these changes can rapidly decrease in a short period of time. One potential explanation for these changes is the influence that external social trends can have on mental health, such as implementation of broad social policies related to epidemic control (2, 3), communications from media (27), health experts, and political leadership. Future studies should focus not only on the description of the mental health consequences, but also in establishing evidence of possible causal relationships between the dynamic of the disease, public health policies and mental health indicators. For example, it would be important to tease apart the effects of fear of disease, subjective and objective aspects of social isolation, economic uncertainty, and other challenges to mental health.

## Data Availability Statement

The raw data supporting the conclusions of this article will be made available by the authors on request, contingent on approval of the University of Waterloo Office of Research Ethics.

## Ethics Statement

The studies involving human participants were reviewed and approved by the University of Waterloo's Research Ethics Committee (ORE#42932). The patients/participants provided their written informed consent to participate in this study.

## Author Contributions

JH, CP, NH, and PH designed the study. JH and RA analyzed the data. GB wrote the first draft of the manuscript. All authors discussed the ideas and commented on subsequent drafts of the manuscript.

## Conflict of Interest

The authors declare that the research was conducted in the absence of any commercial or financial relationships that could be construed as a potential conflict of interest.

## References

[1] Wang C Chudzicka-Czupała A Tee ML Núñez MIL Tripp C Fardin MA. A chain mediation model on COVID-19 symptoms and mental health outcomes in Americans, Asians and Europeans. Sci Rep. (2021) 11:6481. 10.1038/s41598-021-85943-7 33742072 PMC7979938

[2] Tran BX Nguyen HT Le HT Latkin CA Pham HQ Vu LG. Impact of COVID-19 on economic well-being and quality of life of the vietnamese during the national social distancing. Front Psychol. (2020) 11:565153. 10.3389/fpsyg.2020.565153 33041928 PMC7518066

[3] Wang C Chudzicka-Czupała A Grabowski D Pan R Adamus K Wan X. The association between physical and mental health and face mask use during the COVID-19 pandemic: a comparison of two countries with different views and practices. Front Psychiatry. (2020) 11:569981. 10.3389/fpsyt.2020.569981 33033485 PMC7510452

[4] Brooks SK Webster RK Smith LE Woodland L Wessely S Greenberg N. The psychological impact of quarantine and how to reduce it: rapid review of the evidence. Lancet. (2020) 395:912–920. 10.1016/S0140-6736(20)30460-8 32112714 PMC7158942

[5] Kikuchi H Machida M Nakamura I Saito R Odagiri Y Kojima T. Changes in psychological distress during the COVID-19 pandemic in Japan: a longitudinal study. J Epidemiol. (2020) 30:522–8. 10.2188/jea.JE20200271 32963212 PMC7557175

[6] Solomou I Constantinidou F. Prevalence and predictors of anxiety and depression symptoms during the COVID-19 pandemic and compliance with precautionary measures: age and sex matter. Int J Environ Res Public Health. (2020) 17:4924. 10.3390/ijerph17144924 32650522 PMC7400373

[7] Wang C Pan R Wan X Tan Y Xu L Ho CS. immediate psychological responses and associated factors during the initial stage of the 2019 coronavirus disease (COVID-19) epidemic among the general population in China. Int J Environ Res Public Health. (2020) 17:1729. 10.3390/ijerph17051729 32155789 PMC7084952

[8] Qiu J Shen B Zhao M Wang Z Xie B Xu Y. A nationwide survey of psychological distress among Chinese people in the COVID-19 epidemic: implications and policy recommendations. Gen Psychiatry. (2020) 33:e100213: 10.1136/gpsych-2020-100213 32215365 PMC7061893

[9] Moccia L Janiri D Pepe M Dattoli L Molinaro M De Martin V. Affective temperament, attachment style, and the psychological impact of the COVID-19 outbreak: an early report on the Italian general population. Brain Behav Immun. (2020) 87:75–9. 10.1016/j.bbi.2020.04.048 32325098 PMC7169930

[10] Xiong J Lipsitz O Nasri F Lui LMW Gill H Phan L. Impact of COVID-19 pandemic on mental health in the general population: a systematic review. J Affect Disord. (2020) 277:55–64. 10.1016/j.jad.2020.08.001 32799105 PMC7413844

[11] Pieh C Budimir S Delgadillo J Barkham M Fontaine JRJ Probst T. Mental health during COVID-19 lockdown in the United Kingdom. Psychosom Med. (2021) 83:328–37. 10.1097/PSY.0000000000000871 33009276

[12] Wang C Tee M Roy AE Fardin MA Srichokchatchawan W Habib HA. The impact of COVID-19 pandemic on physical and mental health of Asians: a study of seven middle-income countries in Asia. PloS ONE. (2021) 16:e0246824. 10.1371/journal.pone.0246824 33571297 PMC7877638

[13] Serafini G Parmigiani B Amerio A Aguglia A Sher L Amore M. The psychological impact of COVID-19 on the mental health in the general population. QJM. (2020) 113:531–7. 10.1093/qjmed/hcaa201 32569360 PMC7337855

[14] Hirdes JP van Everdingen C Ferris J Franco-Martin M Fries BE Heikkilä J. The interRAI suite of mental health assessment instruments: an integrated system for the continuum of care. Front Psychiatry. (2020) 10:926. 10.3389/fpsyt.2019.00926 32076412 PMC6978285

[15] Sedigh G Devlin RA Grenier G. Are quebecers more stressed out at work than others? An investigation into the differences between Quebec and the rest of Canada in level of work stress. Can Public Policy. (2017) 43:177–89. 10.3138/cpp.2016-068

[16] Dilmaghani M Tabvuma V. Fragile Families in Quebec and the Rest of Canada: a comparison of parental work-life balance satisfaction. Popul Res Policy Rev. (2021) 1–34. 10.1007/s11113-021-09649-4

[17] Salari N Hosseinian-Far A Jalali R Vaisi-Raygani A Rasoulpoor S Mohammadi M. Prevalence of stress, anxiety, depression among the general population during the COVID-19 pandemic: a systematic review and meta-analysis. Glob Health. (2020) 16:57. 10.1186/s12992-020-00589-w 32631403 PMC7338126

[18] Groarke JM Berry E Graham-Wisener L McKenna-Plumley PE McGlinchey E Armour C. Loneliness in the UK during the COVID-19 pandemic: Cross-sectional results from the COVID-19 Psychological Wellbeing Study. PLoS ONE. (2020) 15:e0239698. 10.1371/journal.pone.0239698 32970764 PMC7513993

[19] Berry I Soucy J-PR Tuite A Fisman D. Open access epidemiologic data and an interactive dashboard to monitor the COVID-19 outbreak in Canada. CMAJ. (2020) 192:E420–E420. 10.1503/cmaj.75262 32392510 PMC7162433

[20] Wickham H. ggplot2. WIREs Comput Stat. (2011) 3:180–5. 10.1002/wics.147

[21] Fitzpatrick KM Harris C Drawve G. Fear of COVID-19 and the mental health consequences in America. Psychol Trauma. (2020) 12:S17–21. 10.1037/tra0000924 32496100

[22] Galindo-Vázquez O Ramírez-Orozco M Costas-Muñiz R Mendoza-Contreras LA Calderillo-Ruíz G Meneses-García A. Symptoms of anxiety, depression and self-care behaviors during the COVID-19 pandemic in the general population. Gac Med Mex. (2020) 156:298–305. 10.24875/GMM.20000266 32831341 PMC8327400

[23] Fullana MA Hidalgo-Mazzei D Vieta E Radua J. Coping behaviors associated with decreased anxiety and depressive symptoms during the COVID-19 pandemic and lockdown. J Affect Disord. (2020) 275:80–1. 10.1016/j.jad.2020.06.027 32658829 PMC7329680

[24] Canet-Juric L Andrés ML del Valle M López-Morales H Poó F Galli JI. A longitudinal study on the emotional impact cause by the COVID-19 Pandemic quarantine on general population. Front Psychol. (2020) 11:565688. 10.3389/fpsyg.2020.565688 33071893 PMC7531077

[25] Wang C Pan R Wan X Tan Y Xu L McIntyre RS. A longitudinal study on the mental health of general population during the COVID-19 epidemic in China. Brain Behav Immun. (2020) 87:40–8. 10.1016/j.bbi.2020.04.028 32298802 PMC7153528

[26] Cao W Fang Z Hou G Han M Xu X Dong J. The psychological impact of the COVID-19 epidemic on college students in China. Psychiatry Res. (2020) 287:112934. 10.1016/j.psychres.2020.112934 32229390 PMC7102633

[27] Gao J Zheng P Jia Y Chen H Mao Y Chen S. Mental health problems and social media exposure during COVID-19 outbreak. PLoS ONE. (2020) 15:e0231924. 10.1371/journal.pone.0231924 32298385 PMC7162477

[28] Phillips CA Caldas A Cleetus R Dahl KA Declet-Barreto J Licker R. Compound climate risks in the COVID-19 pandemic. Nat Clim Change. (2020) 10:586–8. 10.1038/s41558-020-0804-2

[29] Soh HL Ho RC Ho CS Tam WW. Efficacy of digital cognitive behavioural therapy for insomnia: a meta-analysis of randomised controlled trials. Sleep Med. (2020) 75:315–325. 10.1016/j.sleep.2020.08.020 32950013

[30] Bokolo AJ. Exploring the adoption of telemedicine and virtual software for care of outpatients during and after COVID-19 pandemic. Ir J Med Sci. (2021) 190:1–10. 10.1007/s11845-020-02299-z 32642981 PMC7340859

[31] Tzeng N-S Chung C-H Chang C-C Chang H-A Kao Y-C Chang S-Y. What could we learn from SARS when facing the mental health issues related to the COVID-19 outbreak? A nationwide cohort study in Taiwan. Transl Psychiatry. (2020) 10:1–9. 10.1038/s41398-020-01021-y 33024072 PMC7538046

[32] McFarlane AC Hooff MV. Impact of childhood exposure to a natural disaster on adult mental health: 20-year longitudinal follow-up study. Br J Psychiatry. (2009) 195:142–148. 10.1192/bjp.bp.108.054270 19648546

[33] Alexandra ITSP John PH Leslie AE George AH Philip LB. Using standard clinical assessments for home care to identify vulnerable populations before, during, and after disasters. J Emerg Manag. (2017) 15:355–66. 10.5055/jem.2017.0344 29308597

[34] Escobar GJ Adams AS Liu VX Soltesz L Chen Y-FI Parodi SM. Racial Disparities in COVID-19 Testing and Outcomes. Ann Intern Med. (2021) 174:786–93. 10.7326/M20-6979 33556278 PMC7893537

[35] Hsiao V Chandereng T Lankton RL Huebner JA Baltus JJ Flood GE. Disparities in telemedicine access: a cross-sectional study of a newly established infrastructure during the COVID-19 pandemic. Appl Clin Inform. (2021) 12:445–58. 10.1055/s-0041-1730026 34107542 PMC8189758

[36] Thompson ME Forbes WF. The problem of low response rates in surveys of the elderly. Math Sci. (1989) 14:127–37.

[37] Jobe JB Mingay DJ. Cognitive laboratory approach to designing questionnaires for surveys of the elderly. Public Health Rep. (1990) 105:518–24. 2120731 PMC1580104

[38] Thorslund M Wärneryd B. Surveying the elderly about health, medical care and living conditions. Some issues of response inconsistency. Arch Gerontol Geriatr. (1990) 11:161–73. 10.1016/0167-4943(90)90009-U 15374488

[39] Lawton MP. Emotion in Later Life. Curr Dir Psychol Sci. (2001) 10:120–3. 10.1111/1467-8721.00130

[40] Wight RG SepÚlveda JE Aneshensel CS. Depressive symptoms: how do adolescents compare with adults? J Adolesc Health. (2004) 34:314–23. 10.1016/j.jadohealth.2003.05.003 15041001

